# Recurrent Unprovoked Deep Vein Thromboses in the Setting of Sarcoidosis

**DOI:** 10.7759/cureus.18528

**Published:** 2021-10-06

**Authors:** Brinda Basida, Maryam B Haider, Joshua Barbosa

**Affiliations:** 1 Internal Medicine, Detroit Medical Center Sinai-Grace Hospital, Detroit, USA; 2 Internal Medicine, Detroit Medical Center, Wayne State University, Sinai-Grace Hospital, Detroit, USA; 3 Kresge Eye Institute, Detroit Medical Center, Wayne State University, Detroit, USA

**Keywords:** deep venous thrombosis (dvt), unprovoked venous thromboembolism, unprovoked thromboembolism, venous thromboembolism (vte), rheumatology & autoimmune diseases, pulmonary sarcoidosis

## Abstract

Sarcoidosis is described as a state of immune-mediated chronic systemic inflammatory disease that is typically characterized by non-caseating granulomas. It involves multiple organs like the lungs, lymph nodes, eyes, skin, and liver. Besides the solid organ involvement, it is also known to be associated with various pro-thrombotic states leading to pulmonary embolism and deep vein thrombosis. Mechanical factors causing the venous stasis were considered the major contributing factors. However, based on recent studies, chronic inflammation from macrophages and activated leukocytes are also hypothesized to further activate thrombin and fibrin formation. Regardless of the etiology, the management focuses on life-long anticoagulation to prevent further episodes of thrombosis. Herein we present a case of a 38-year-old male with a history of sarcoidosis who was admitted after having recurrent deep vein thromboses. Further investigations revealed a negative hypercoagulable workup or other auto-immune processes and the absence of any granulomas causing mechanical compression on large vessel venous vasculature. He was adequately managed with anticoagulation therapy and followed up outpatient with no further similar episodes. This case adds to the growing understanding that the mechanisms by which sarcoidosis induces thromboembolism is primarily pro-inflammatory rather than mechanical in nature.

## Introduction

Sarcoidosis is an immune-mediated disorder of unknown etiology that is characterized by the presence of non-caseating granulomas. There is a predilection for pulmonary and intrathoracic lymph node involvement with over 90% of sarcoidosis patients having the involvement of the pulmonary system [1-3]. That sarcoidosis patients are at increased risk of thrombosis has been known for some time [4]. A cohort of 345 sarcoidosis cases compared to controls over 37 years suggests that the adjusted hazard ratio of venous thromboembolism after correcting for age and sex was 3.04 (CI95: 1.47-6.29) [4]. The increased risk for thromboembolism in the sarcoidosis population has been attributed to either mechanical factors, wherein the granulomas physically compress veins disrupting normal fluid dynamics, or a pro-inflammatory state tipping the coagulation cascade to prothrombin formation. Herein, we present a case of unprovoked deep vein thromboses in a sarcoidosis patient suggestive of the latter mechanism for hypercoagulability.

## Case presentation

A 38-year-old male with a past medical history of biopsy-proven sarcoidosis from a submandibular lymph node presented to the emergency department with right-sided, medial thigh pain of one-day duration. The pain was localized to the medial aspect of the right thigh, was non-radiating, and worse with activity though present at rest as well. The patient denied fever, chills, a history of hemarthrosis, difficulty breathing, trauma to the leg, any recent travel, and prolonged bed rest. The patient was hemodynamically stable saturating 99% on room air with a heart rate of 82 beats per minute, a temperature of 36.8 degrees Celsius, and blood pressure of 128/90 mmHg. Physical exam demonstrated tenderness to palpation over the medial aspect of the right thigh but no erythema or warmth. Past medical history was remarkable for sarcoidosis and recurrent episodes of deep vein thrombosis. The patient had two prior episodes both considered being provoked. The first episode in 2005 resulted in the placement of an inferior vena cava filter. Surgical and medical history was otherwise unremarkable. The patient endorsed a family history of deep vein thrombosis in his father's side of the family and smoked two packs of cigarettes per day for a 20-year duration. Lower extremity venous duplex ultrasound suggested “acute deep vein thrombosis involving the common femoral, saphenofemoral junction, femoral and popliteal veins” on the right lower extremity and “subacute deep vein thrombosis involving the common femoral, saphenofemoral junction, femoral and popliteal veins” in the left lower extremity.

**Figure 1:**
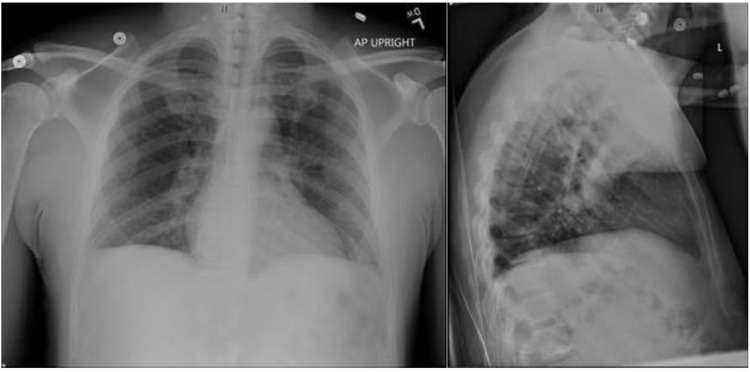
Chest X-Ray of Patient With Lymph Node Biopsy Demonstrating Sarcoidosis

While admitted, a chest X-ray and angiotensin-converting enzyme (ACE) levels were obtained to evaluate the extent of sarcoidosis activity. ACE levels were 108 U/L (normal range 9 to 67) and the chest X-ray did not show evidence of peri-hilar or mediastinal lymphadenopathy (Figure 1). Given the recurrence of deep vein thrombosis (DVT) in the setting of a family history of recurrent DVTs, a basic coagulopathy workup was obtained. Fibrinogen, factor IX, factor XI, and factor XIII activity were normal. Antinuclear antibody (ANA) fluorescent screen, beta 2 glycoprotein screen, cardiolipin antibody screen, syphilis enzyme immunoassay (EIA) screen, and lupus screen resulted as none detected. Factor VIII activity was 226% (normal 60% to 168%) and Von Willebrand activity was 139% (upper limit of normal 138%). Genotyping demonstrated that the patient was not a carrier of the 1691 G>A mutation, colloquially the factor V Leiden mutation. ADAMTS13 activity was 80% (normal 75 to 117%).

Based on laboratory and ultrasound findings, a diagnosis of recurrent deep vein thrombosis was made. This was attributed to the underlying pro-inflammatory mechanism of sarcoidosis in the absence of structural granulomas that would impede normal fluid dynamics of the inferior vena cava. This case caused one of the most difficult conundrums for the diagnosis of the underlying mechanism of recurrent DVTs in sarcoidosis. HIV and syphilis infection was ruled out based on the serology. Other autoimmune conditions like systemic lupus erythematosus (SLE) and antiphospholipid syndrome were also ruled out based on immunology results and lack of supportive clinical findings. Genetic diseases of factor V Leiden mutation, ADAMTS13 mutation, and hemophilia were excluded from the laboratory results. Furthermore, elevated factor VIII and von Willebrand activity favor the intrinsic hypercoagulable state in this patient.

The patient was started on a heparin drip prior to bridging him to warfarin. He was then continued on warfarin treatment with clinical improvement. The patient’s symptoms improved and were instructed to follow-up outpoint at our INR clinic. He did so and continues to receive appropriate monitoring of his INR without subsequent recurrence of deep vein thrombosis or pulmonary embolisms.

## Discussion

The underlying reason why sarcoidosis patients are predisposed to venous thromboembolism remains incompletely understood. Virchow’s triad of venous stasis, endothelial injury, and increased blood coagulability provides a conceptual model for underlying causal factors that may contribute to hypercoagulable states. A record link study out of the United Kingdom reported a significantly elevated risk of DVTs and PEs in 23 autoimmune disorders, which suggest the hypercoagulable state may be related to inflammatory processes [5]. Rebeiz et al. have reported a case of a 32-year-old male with sarcoidosis who developed a pulmonary embolism with a thrombophilia profile consisting of factor II, MTHFR, and factor XIII gene mutations [6]. This patient had resulting elevations in homocysteine levels [6]. Our case presentation portrays the possibility that sarcoidosis can cause an inflammatory hypercoagulable state that antedates the presence of a clinically evident disease.

Various chronic inflammatory states like vasculitis, rheumatoid arthritis, and SLE have been recognized as risk factors for venous thromboembolism (VTE) [4,7-9]. Endothelial cells, leukocytes, and platelets are activated to form microparticles that trigger an increased activation of thrombin and fibrin formation [9-10], which ultimately results in a thrombotic state as characterized by abnormal tissue thromboplastin activity and D-dimer levels. Decreased plasminogen activator activity and protein C activation are a few of the other abnormalities [11]. Studies have shown that circulating molecules like interleukins IL-1β, IL-6, and IL-8 can cause hypercoagulation and hypofibrinolysis by directly forming fibrin clots from plasma cells, erythrocytes, and platelets and thus play a major role in acute and chronic inflammation [12].

Several other factors can potentially contribute to the development of VTE in sarcoidosis besides the inflammatory factors. These include the chronic use of glucocorticoids, immobilization from the disabling disease, and the presence of additional autoimmune conditions (antiphospholipid antibodies were detected in 38% of sarcoidosis patients in one study) [13]. Our patient did not have any underlying remarkable autoimmune conditions. This case adds growing evidence to the mechanism of hypercoagulability being intrinsic in nature versus mechanical.

## Conclusions

We present a case of a 38-year-old with recurrent, unprovoked DVTs with non-significant elevated factor VIII and von Willebrand activity in the absence of structural granulomas that would impede normal fluid dynamics of the inferior vena cava. This case adds growing evidence to the mechanism of hypercoagulability being intrinsic in nature versus mechanical. Patients with an autoimmune disease and a diagnosis of sarcoidosis are predisposed to unprovoked VTEs. In sarcoidosis patients with unprovoked DVTs, underlying coagulation defects are not unheard of.
